# Long Non-coding RNA 332443 Inhibits Preadipocyte Differentiation by Targeting Runx1 and p38-MAPK and ERK1/2-MAPK Signaling Pathways

**DOI:** 10.3389/fcell.2021.663959

**Published:** 2021-06-08

**Authors:** Fen Xiao, Chen-Yi Tang, Hao-Neng Tang, Hui-Xuan Wu, Nan Hu, Long Li, Hou-De Zhou

**Affiliations:** ^1^National Clinical Research Center for Metabolic Diseases, Hunan Provincial Key Laboratory of Metabolic Bone Diseases, and Department of Metabolism and Endocrinology, The Second Xiangya Hospital of Central South University, Changsha, China; ^2^Department of Laboratory Medicine, The Second Xiangya Hospital, Central South University, Changsha, China

**Keywords:** lncRNA, adipogenic differentiation, 3T3-L1 preadipocytes, Runx1, MAPK

## Abstract

Long non-coding RNAs (lncRNAs) have emerged as integral regulators of pathophysiological processes, but their specific roles and mechanisms in adipose tissue development remain largely unknown. Here, through microarray analysis, co-expression, and tissue specific analysis of adipocyte tissues after fasting for 72 h, we found that *Lnc-FR332443* expression was dramatically decreased, as well as the expression of *Runx1*. The UCSC database and Ensembl database indicated that *Lnc-FR332443* is the antisense lncRNA of *Runx1*. *Lnc-FR332443* and *Runx1* are highly enriched in adipose tissue and downregulated during adipogenic differentiation. Adipose tissue-specific knockdown of *Lnc-FR332443* increased fat mass *in vivo*, and specific knockdown of *Lnc-FR332443* in 3T3-L1 preadipocytes promoted adipogenic differentiation. In this process, *Runx1* expression was decreased when *Lnc-FR332443* was downregulated in adipocytes or 3T3-L1 preadipocytes, and vice versa, when *Lnc-FR332443* was upregulated, the expression of *Runx1* was increased. However, overexpression of *Runx1* decreased the expression of the adipocyte cell marker genes *PPAR*γ, *C/EBP*α and *FABP4* significantly, while not affected the expression of *Lnc-FR332443.* Mechanistically, *Lnc-FR332443* positively regulates *Runx1* expression in mouse adipocytes and suppresses adipocyte differentiation by attenuating the phosphorylation of MAPK-p38 and MAPK-ERK1/2 expression. Thus, this study indicated that *Lnc-FR332443* inhibits adipogenesis and which might be a drug target for the prevention and treatment of obesity.

## Introduction

Obesity is related to a series of metabolic abnormalities and has become an international public health issue that affects quality of life, increases the risk of diseases and shortens life expectancy. However, the specific mechanism of the occurrence and development of obesity is still unclear, and there are few effective drug targets.

Long non-coding RNAs (lncRNAs) are a class of ncRNAs transcribed from the genome that do not encode proteins and are longer than 200 nt (Mercer et al., 2009). Numerous lncRNAs are involved in the regulation of important genes and cell fate (Wahlestedt, 2013; Fatica and Bozzoni, 2014). Moreover, lncRNAs are widely involved in the occurrence and development of tumors (Huarte, 2015; Schmitt and Chang, 2016). In recent years, lncRNAs have received increasing attention as new drug targets for obesity. Some studies have proven that non-coding RNAs play an important role in the occurrence and development of obesity (Ponting et al., 2009). LncRNAs are involved in the adipogenesis and development of white and brown adipose tissues (Alvarez-Dominguez et al., 2015; Bai et al., 2017; Li et al., 2017; You et al., 2018). In addition, some lncRNAs have been evaluated as biomarkers and drug targets for the diagnosis and treatment of obesity (Ballantyne et al., 2016; Cui et al., 2016). However, to date, the specific regulatory mechanism of lncRNAs in adipogenic differentiation is still unclear, and there are few lncRNAs that can be used as targets of anti-obesity drugs.

*Runx1*, a member of the Runx (transcription factor associated with runt) family, has been shown to regulate the differentiation of hematopoietic stem cells into mature blood cells (Cai et al., 2015; Guo et al., 2020). In addition, *Runx1* may play an important role in the phenotype maintain and differentiation of osteoblasts and chondrocyte lineages (LeBlanc et al., 2015; Tang J. et al., 2020). In our previous research, we found that the specific deletion of *Runx1* in osteoblasts and chondrocytes promotes adipogenic differentiation, suggesting that *Runx1* can cell autonomously and cell non-autonomously regulate adipocyte differentiation (Tang C.Y. et al., 2020; Tang et al., 2021). However, the pathway upstream or downstream of *Runx1* that causes inhibition of adipocyte differentiation remains unclear.

In this study, we identified a metabolism-sensitive lncRNA (named *Lnc-FR332443*) which was enriched in adipose tissue, regulated by external specific nutritional stimulation and downregulated during adipogenic differentiation *via* high-throughput microarray screening. In mice with adipose tissue-specific knockdown of *Lnc-FR332443* by AAV-Lnc-FR332443-siRNA, whose fat mass was increased. Interestingly, *Runx1* expression decreased significantly in mesenteric white adipose tissue (mWAT), subcutaneous white adipose tissue (sWAT), and intrascapular brown adipose tissue (iBAT) in these mice. With adipogenic differentiation of 3T3-L1 preadipocytes *in vitro*, *Lnc-FR332443* and *Runx1* were significantly downregulated. Knockdown the expression of *Lnc-FR332443* increased the expression of *Runx1* and the adipogenic differentiation of 3T3-L1 cells. Mechanistically, *Lnc-FR332443* upregulated the expression of *Runx1* in mouse adipocytes and thus epigenetically inhibited adipocyte differentiation, which might be achieved by attenuating the phosphorylation of p38 and ERK1/2. Our study revealed the function and mechanism of *Lnc-FR332443* in the negative regulation of adipogenesis and suggested that *FR332443* might be a potential strategy for the prevention and treatment of obesity.

## Materials and Methods

### Ethics Statement

The animal study was carried out under the approval and supervision of the ethics committee of the Second Xiangya Hospital, Central South University.

### Animal Experiments

Three-month-old C57BL/6J male mice were purchased from the Model Animal Research Center of Central South University, and all animal experimental procedures were approved by the Animal Care Committee of Central South University (Changsha, China). C57BL/6J male mice were housed individually in ventilated Plexiglas cages in climate-controlled quarters (22°C ∼ 25°C) in a pathogen-free barrier facility and maintained under a 12-h light/dark cycle with standard rodent chow (7.0% fat, 18.7% protein, 64.7% carbohydrates, and 5.0% fiber). Diets were supplemented with a similar mix of minerals and vitamins according to the standards of the American Institute of Nutrition (AIN-93G), and the animals were acclimatized to housing conditions for at least 7 days before the experiments.

### Microarray Analysis

Microarray analysis was performed by OeBiotech Corporation (Shanghai, China). An Agilent mouse lncRNA microarray (4 × 180 K, Design ID: 049801) with 33,420 mRNAs and 54,030 lncRNAs transcripts was used in this experiment. RNA integrity in TRIzol reagent was assessed using an Agilent Bioanalyzer 2100 (Agilent Technologies, CA, United States). Sample labeling, microarray hybridization, and washing were performed based on the manufacturer’s standard protocols. After cRNA was hybridized to the microarray, the array was scanned after cleaning with an Agilent G2505C scanner (Agilent Technologies, CA, United States). GeneSpring (version 13.1, Agilent Technologies, CA, United States) was used to finish the basic analysis with the raw data. Differentially expressed genes or lncRNAs were then identified through fold changes as well as *P* values calculated with *t*-tests. The threshold set for up- and downregulated genes was a fold change ≥ 2.0 and a *P* value ≤ 0.05. Through hierarchical clustering, the gene expression patterns between samples can be distinguished. With the help of Cytoscape 3.11 (Agilent and IBS), diagrams of the lncRNA-mRNA network and lncRNA-TF network were drawn for co-expression network analysis.

### Gene Ontology Analysis and Pathway Analysis

Gene ontology (GO)^1^ (a functional analysis utilizing GO categories) analysis was used to describe the function of differentially expressed lncRNAs and co-expressed mRNAs. Additionally, we used the KEGG database^2^ to identify significant pathways for predicted target genes. GO term enrichment and the biological pathways utilized significant *P*-values (recommended *P*-value < 0.05).

### Cell Lines Culture and Adipocyte Differentiation

We obtained 3T3-L1 preadipocytes from the American Type Culture Collection (ATCC, United States). Cells were cultured in Dulbecco’s modified Eagle’s medium (Thermo Fisher Scientific, DMEM, Gibco, MA, United States) containing 10% fetal bovine serum (FBS; Thermo Fisher Scientific, Gibco, MA, United States), 100 mg/ml penicillin and 50 μg/ml streptomycin (Thermo Fisher Scientific, Gibco, MA, United States) in an incubator with 5% CO_2_ and 37°C atmosphere. The 3T3-L1 preadipocytes were passaged at 70–80%, seeded in 24-well dishes and induced to differentiate (day 0) 2 days after the cells were grown to complete fusion for contact inhibition. Induction was performed using a classic cocktail regimen: 0.5 mmol/L IBMX, 10^–6^ mol/L Dex and 5 μg/mL insulin. After 2 days, complete medium containing only 5 μg/mL insulin was added for 48 h, the culture was continued in complete medium without any inducer, and the medium was refreshed every 2 days. Up to 90% of the cells had been differentiated into mature adipocytes by day 10.

### HE Staining and Adipocyte Size Measurements

Adipose tissue specimens were weighed and individually placed in a 4% paraformaldehyde solution and fixed at 4°C for 24 h. After washing with PBS, paraffin embedding was used to make 5 μm paraffin sections, and the samples were stained with H&E for morphological observation. An optical microscope with digital camera (Olympus, Japan) was used to collect adipocytes. Three to five samples were collected randomly under each tissue microscope, and then, all images were scanned and counted by Image-Pro Plus software to calculate the average area of adipocytes. Cell areas (μm^2^) were measured and averaged for each section.

### Oil Red O Staining

Oil red O (ORO) dyeing solution (Zhuhai Beso Biotechnology Co., Ltd., China) was added to double-distilled water at a 3:2 ratio, mixed evenly, and then filtered twice with filter paper. Cultured 3T3-L1 preadipocytes at differentiation days 0, 2, 4, 6, 8, and 10 were washed twice with PBS, fixed with 4% paraformaldehyde at room temperature for 20–30 min, washed, added to 1 mL of filtered oil red O working solution and stained at room temperature for 2 h in the dark. Then, the excess staining solution was washed out, and PBS liquid was added to cover the cell surface. The staining was observed under an inverted microscope (Nikon, Japan), and pictures were taken.

### Plasmid Transfection and Lentivirus Experiments

Knockdown of *Lnc-FR332443* expression was performed with LncRNA Smart Silencer (RiboBio; Guangzhou, China). LncRNA Smart Silencer transfection was performed using Lipofectamine 3000 (Invitrogen; Carlsbad, CA, United States) at a concentration of 100 nM. At least 24 h before transfection, 3T3-L1 preadipocytes were added to 6-well plates at a density of 2.5 × 10^5^ cells/well to achieve 40–50% confluency. For MAPK signaling pathway, Bmp7 (50 ng/ml, Sigma-Aldrich, St. Louis, United States) was used to stimulate 3T3-L1 preadipocytes at different time points. The cells were collected for RNA isolation or protein extraction after transfection for 24 h. The *Lnc-FR332443* overexpression construct was cloned into a lentiviral expression vector for packaging viruses, and the lentivirus was amplified in HEK293T cells and concentrated using polyethylene glycol (System Biosciences, CA, United States). For overexpression experiments, subconfluent 3T3-L1 preadipocytes were infected, and the infection efficiency was confirmed by quantitative PCR (qPCR). Cells were collected for RNA isolation after stable transfection for 72 h.

### Adenoviral Vector Construction and Purification

Adenoviral vectors encoding *Lnc-FR332443-siRNA* or *Runx1* were generated by the AAV Helper-Free System. Adenovirus was produced in 3T3-L1 preadipocytes or HEK 293T cells and purified by the Add-N-Pure Adenovirus Purification Kit (Abcam, Cambridge, MA, United States) according to the manufacturer’s instructions (HanBio, Shanghai, China). After AAV infection, the total RNA of cells was extracted for qRT-PCR detection or Western blotting efficiency identification.

### Quantitative Real-Time PCR

Total RNA was extracted from tissue samples or cells using TRIzol Reagent according to the manufacturer’s instructions (Thermo Fisher Scientific, MA, United States). Reverse transcription was performed to synthesize cDNA using an RT Kit (TaKaRa, Otsu, Japan). The primers used for real-time qRT-PCR are shown in Table 1. qRT-PCR was performed on a Light Cycler 480 Real-time PCR Detection System (Roche, Basel, Switzerland) using SYBR Green PCR master mix (TaKaRa, Otsu, Japan) following the manufacturer’s instructions. Relative lncRNA or mRNA expression was normalized to that of GAPDH and is expressed as 2^–Δ^ ^Δ^ ^*CT*^ relative to the control group.

**TABLE 1:** Real-time RT-PCR primer sequences.

**Gene**	**Forward primer (5′ to 3′)**	**Reverse primer (5′ to 3′)**
GAPDH	CAATGACCCCTTCATTGACC	GACAAGCTTCCCGTTCTCAG
*Ppar*γ	ATGGTTGACACAGAGATGC	GAATGCGAGTGGTCTTCC
*C/ebp*α	CAAGAACAGCAACGAGTACCG	GTCACTGGTCAACTCCAGCAC
*Fabp4*	AGCACCATAACCTTAGATGGGG	CGTGGAAGTGACGCCTTTCA
*Runx1*	TTTCGCAGAGCGGTGAAAGA	GCACTGTGGATATGAAGGAA
*LncRNA-FR332443*	AAACCCGACAATGTAAGGACC	CACCATTGGGAGGATGTCAG
*LncRNA-ENSMUST0000128000 LncRNA-ENSMUST00000137343 LncRNA-FR067374 LncRNA-FR099179 LncRNA-FR265215 LncRNA-FR289864 LncRNA-FR016359 LncRNA-FR122880 LncRNA-NON-MMUT006554 LncRNA-NON-MMUT015663 LncRNA-NR_027820.1*	ACAATGCCTTGGTCACTGTCAC TTGATGAAGATTGTGAGGAGGG GGGAGGATTCTGTTGCGGTT GGACAGACATGCCATACAGAGG GCACTGAGAGGTTGAAACAAGAG ATGATTCAGAGCGGAGAACTTAGC CCTCTGTTTTAGCACACTCCTCA TTACAAGGCTGGGCATCAAA ATCTTGTTGAAAATCTCAGCGG TTGGAACGAAGGGTCTGGC GAAGCGAGCTGAGAGGCTTT	TCTGGTCTCCCTCCTCACAATC CGGGTAGTGCAACTAAACGG CCTGTTGTCTTCAAGGCTGGT GGGGAGCTTTGTACTTACAAGTGT AAACCTACAGGACCGCATTCT GGATATGGATAGCATTCTTCGG ACTTTTTGGAGGGAGCCGG GACAGATGTGAGACGCTTCCAG GTTGGGTTTAGATGAATGTGGG TGAGCGAGGAATAGGCGAAG GGGGAAACCATGGCATTCTG

### Western Blot Analysis

Protein was extracted from cell lysates using RIPA buffer and quantified by the BCA Protein Assay Kit (Thermo Fisher Scientific, MA, United States). Approximately 30 μg of protein extracts were separated by a 10% SDS-PAGE gel, transferred to a PVDF nitrocellulose membrane (Millipore, MA, United States) for 120 min at 300 mA, blocked with 5% non-fat milk and then incubated with primary antibodies. Antibodies against Runx1 (1:1,000, Abcam, Cambridge, MA, United States), PPARγ (#2,435; 1:1,000, Cell Signaling Technology, Danvers, MA, United States), FABP4 (ab92501, 1:1,000, Abcam, Cambridge, MA, United States), GAPDH (1:1,000, Servicebio, Wu Han, China), phospho-p44/42 MAPK (Erk1/2)(#4,370, Cell Signaling Technology, Danvers, MA, United States), p38 MAPK (#9,212, Cell Signaling Technology, Danvers, MA, United States), phospho-p38 MAPK (#4,631, Cell Signaling Technology, Danvers, MA, United States), phospho-SAPK/JNK (#9,251, Cell Signaling Technology, Danvers, MA, United States) and SAPK/JNK (#9,258, Cell Signaling Technology, Danvers, MA, United States) were used. Secondary blotting was performed using horseradish peroxidase-linked anti-rabbit IgG 163 (7074) and horseradish peroxidase-linked anti-mouse IgG (7,076) (Cell Signaling Technology, Danvers, MA, United States). The bands were visualized using a chemiluminescent peroxidase substrate (Millipore, MA, United States). All measurements were repeated three times. The relative optical density was measured with ImageJ software.

### Statistical Analysis

Statistical analysis was performed by using SPSS software (version 22.0; IBM, CA, United States) or GraphPad Prism (version 8; GraphPad Software, Inc., San Diego, CA, United States). All values are presented as the mean ± SEM, unless otherwise indicated. Statistical analyses consisted of two-tailed unpaired Student’s *t*-test and one-way ANOVA with *post hoc* Tukey’s test. *P* < 0.05 was considered to indicate a statistically significant difference.

## Results

### Identification of Mouse *Lnc-FR332443* in Adipocyte Differentiation

**FIGURE 1:**
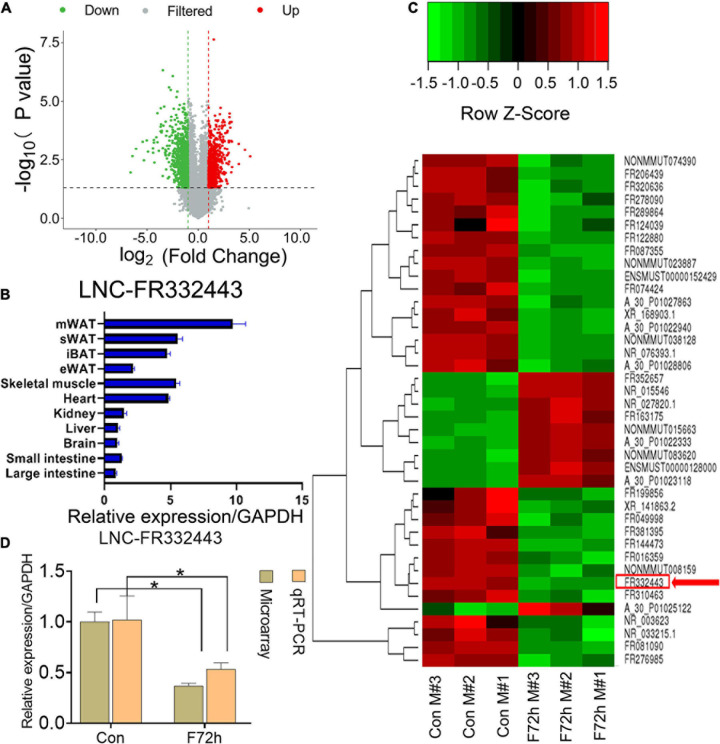
Identification of differentially expressed lncRNAs in F72h mouse mWAT compared with control mouse mWAT. **(A)** A volcano plot illustrating the expression of the differentially regulated lncRNAs from microarray analysis between the control and F72h male mouse mWAT. **(B)** Expression profile of *Lnc-FR332443* across 11 mouse tissues examined by qPCR. **(C)** Cluster heat map showing differentially expressed lncRNAs in mWAT between the F72h mice and control mice. The red arrow shows *Lnc-FR332443*. *N* = 3. **(D)** Comparison of microarray data and qRT-PCR data for *Lnc-FR332443* in mWAT between the F72h mice and the control mice. All data are expressed as the mean ± SEM, *N* = 3, *P < 0.05.

In our previous research, we found that the plasticity of adipose tissues was important for fat distribution after food deprivation or refeeding (Tang et al., 2017). After C57BL/6J male mice were fasted for 72 h (F72h), mWAT mass showed the greatest consumption compared to other regions of adipose tissue (Tang et al., 2017). To further identify lncRNAs selectively involved in adipocyte differentiation, we analyzed differential lncRNAs and mRNAs profiles in mWAT by microarrays, and found 677 downregulated lncRNAs and 513 upregulated lncRNAs (fold > 2, *P* < 0.05) (Figure 1A) among the total 54,030 LncRNAs (Supplementary Material 1) in the F72h mice compared to the control mice. Through differential expression analysis, co-expression analysis (correlation > 0.99, *P* < 0.01), and Venn analysis (Control VS F72), 12 lncRNAs related to adipogenesis, lipid and energy metabolism were selected (Supplementary Figure 1). We conducted qRT-PCR for these 12 lnRNAs expression in mWAT, and found the lncRNA-FR067374, lncRNA-FR265215, lncRNA-FR332443 were all statistically downregulated in F72h, while all obviously upregulated in refeeding for 48 h (R48h) compared to their control groups mWAT (Supplementary Figure 1). Thereafter, we further conducted qRT-PCR for these three lncRNAs in mice tissues, and the results indicated that lncRNA-FR265215 expression level is low in adipose tissues compared with that in other tissues, as well as the expression level of lncRNA-FR067374 (Supplementary Figure 2). However, the expression level of lnc-FR332443 was relatively high in adipose tissues, especially high expressed in mWAT (Figure 1B). As shown in the heatmap, among the top 40 downregulated lncRNAs in the microarray analysis was *lncRNA-FR332443* (Figure 1C, red arrow), which was then selected for further experiments. We further confirmed that *Lnc-FR332443* was significantly downregulated in mWAT of F72h mouse by qRT-PCR, which was consistent with the microarray results (Figure 1D). The results suggested that *lncRNA-FR332443* might play a role in adipocyte differentiation.

### Identification of Mouse *Runx1* Potentially Targeted by *Lnc-FR332443*

**FIGURE 2:**
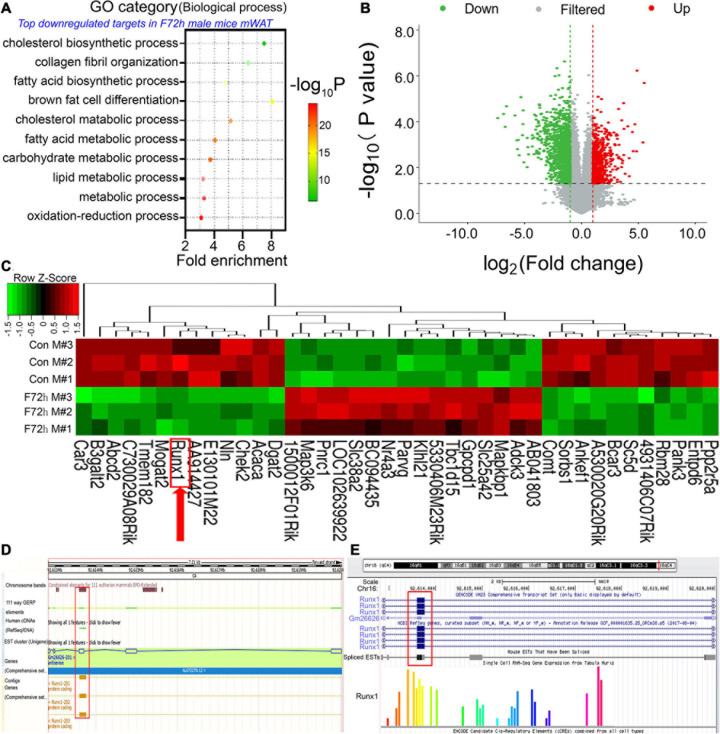
Identification of differentially expressed mRNAs in the F72h mWAT compared to the control mWAT. **(A)** Gene ontology (GO) functional clustering of genes that were downregulated in F72h male mouse mWAT for biological processes (the top significantly affected categories are shown using the KEGG analysis tool). **(B)** A volcano plot illustrating differentially regulated gene expression from RNA-seq analysis between the control and F72h male mouse mWAT. Genes upregulated and downregulated are shown in red and green, respectively. Values are presented as the log2 of tag counts. **(C)** Cluster heat map showing differentially expressed genes in mWAT between the fasted mice and the control mice. The red arrow shows Runx1. *N* = 3. **(D)** UCSC database and **(E)** Ensembl database show that *Lnc-FR332443* is the antisense lncRNA of *Runx1*. *Lnc-FR332443* is also found at Gm26626, and Gm26626-201 is located on chromosome 16: 92612824-92620032. There are five exons in this region, of which exon 3 overlaps with part of the transcripts of the *Runx1* gene (red rectangle).

We analyzed the mRNAs that had significant co-expression with the differentially expressed lncRNAs by GO and KEGG analysis (Figure 2A). The top GO downregulated categories were selected according to the *P*-values and enrichment score and illustrated as the number of genes downregulated in the categories. GO enrichment analysis demonstrated the most significantly affected categories of genes that were downregulated in the F72h mouse mWAT compared to that of the control mice. The top downregulated gene clusters were associated with *fatty acid biosynthetic process*, *fatty acid metabolic process*, *lipid metabolic process* and so on (Figure 2A), which were all closely associated with adipocyte differentiation. To find out the target genes of *lnc-FR332443* in adipocyte differentiation, we analyzed the potential transcription factors of *lnc-FR332443* and found that *Runx1* which had been previously shown in the negative regulation of adipogenesis (Tang et al., 2021) is among them (Supplementary Material 2). Further we analyzed mRNA expression in the F72h mouse and control mouse mWAT using microarrays and measured 1,353 downregulated mRNAs and 494 upregulated mRNAs (fold change > 2, *P* < 0.05) (Figure 2B) among the total 33,420 mRNAs (Supplementary Material 3). As shown in the heatmap, we found that the expression of *Runx1*, the potential target gene of *Lnc-FR332443*, was also significantly downregulated in the microarray analysis (Figure 2C, red arrow). *Lnc-FR332443* is located on chromosome 16:92612824-92620032, and this region contains 5 exons according to the UCSC Genome Browser^3^. The UCSC database (Figure 2D) and Ensembl database (Figure 2E) search results further demonstrated that *Lnc-FR332443* is the antisense lncRNA of *Runx1*. These results indicated that *Lnc-FR332443* might regulate adipocyte differentiation through *Runx1*.

### Increased Fat Mass in Mice With Adipose Tissue-Specific *Lnc-FR332443* Knockdown

**FIGURE 3:**
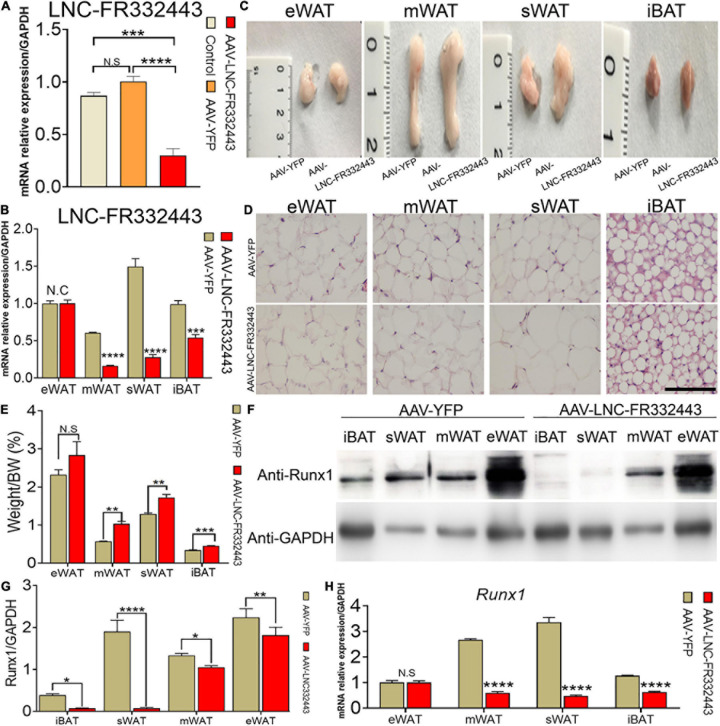
Morphological and functional changes in mouse adipose tissue with specific knockdown of *Lnc-FR332443*. **(A)** The efficiency of *Lnc-FR332443* knockdown by intraperitoneal injection of AAV virus. **(B)** The expression of *Lnc-FR332443* in adipose tissue (eWAT, mWAT, sWAT and iBAT) after wild-type mice were intraperitoneally injected with AAV virus. **(C)** Representative graphs of eWAT, mWAT, sWAT and iBAT from the AAV-Lnc-FR332443 and control mice. **(D)** HE staining for eWAT, mWAT, sWAT and iBAT in the AAV-Lnc-FR332443 mice and control mice. Scale bar, 100 μm. **(E)** The proportion of adipose tissue (eWAT, mWAT, sWAT and iBAT) to total body weight (weight%). **(F)** Western blot analysis of Runx1 expression in different adipose tissues with *Lnc-FR332443* knockdown. **(G)** Quantification data of panel **(F)**. **(H)** qRT-PCR of *Runx1* expression in different adipose tissues with *Lnc-FR332443* knockdown. All data are expressed as the mean ± SEM, N.S denotes not significant, *N* = 3, *P < 0.05, **P < 0.01, ***P < 0.001, and ****P < 0.0001.

To confirm the role of *Lnc-FR332443* in adipogenesis *in vivo*, we administered adenovirus by daily intraperitoneal injection for 21 days to conditionally eliminate the expression of *Lnc-FR332443*. The results showed that *Lnc-FR332443* expression decreased by about 75%, 85%, and 41% in mWAT (Figure 3A), sWAT, and iBAT (Figure 3B) respectively in the AAV-Lnc-FR332443 group compared to the control group, however, no significant difference of *Lnc-FR332443* expression was found in eWAT (Figure 3B). The Lnc-FR332443 knockdown mice displayed a thicker fat pad mass in mWAT, sWAT, and iBAT than their control counterparts (Figure 3C). Consistently, HE staining showed that the size of adipocytes in both mWAT and sWAT was larger in the AAV-Lnc-FR332443 group mice, and the number of adipocytes in iBAT was obviously increased in the AAV-Lnc-FR332443 group mice compared to the control group mice, but there was no significant change for mWAT (Figure 3D) in the AAV-Lnc-FR332443 group compared with its control group mice. The ratios of the sWAT, mWAT, and iBAT weight to the whole body weight were significantly increased in the AAV-Lnc-FR332443 group mice, but no significant difference was found for eWAT (Figure 3E). Under the same experimental conditions, we also found that the expression of *Runx1*, an antisense transcript of *Lnc-FR332443*, was decreased in iBAT, sWAT, mWAT, and eWAT in the AAV-Lnc-FR332443 group mice compared to their controls (Figures 3F,G). In addition, the *Runx1* mRNA level was decreased in iBAT, sWAT, and mWAT in the AAV-Lnc-FR332443 group mice compared to their controls (Figure 3H). However, no difference of *Runx1* mRNA expression was found in eWAT (Figure 3H). Considering the essential functions of *Runx1* in many biological programs related to development (Ichikawa et al., 2004) and our previous research results that *Runx1* could cell autonomously and cell non-autonomously regulate adipocyte differentiation (Tang C.Y. et al., 2020; Tang et al., 2021), we hypothesized that *Runx1* might be involved in the process of fat formation in the WAT of the AAV-Lnc-FR332443 mice.

### Both *Lnc-FR332443* and *Runx1* Were Downregulated During Adipogenesis, and Overexpression of *Lnc-FR332443* Increased the Expression of Runx1

**FIGURE 4:**
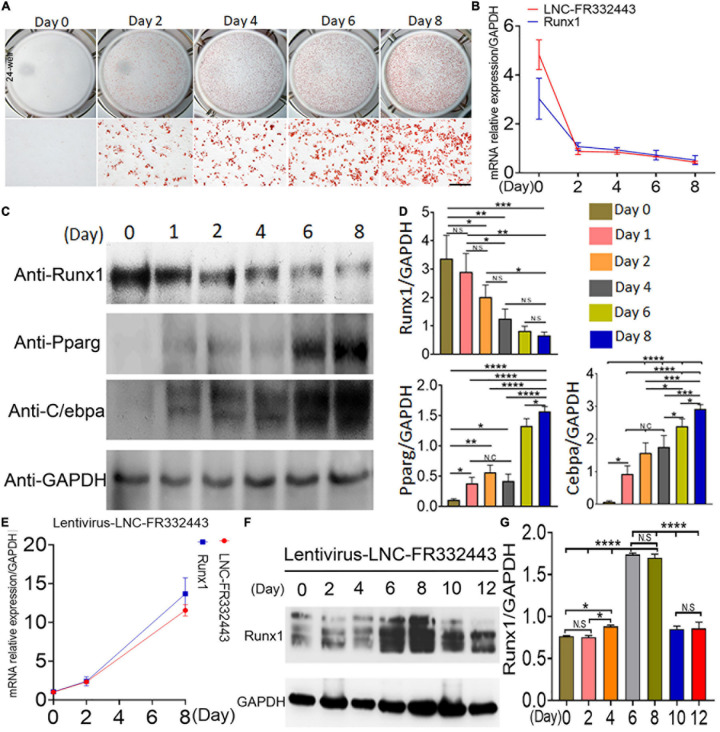
Downregulation of *Lnc-FR332443 and Runx1* expression during adipogenic differentiation. **(A)** Oil red O staining of mouse 3T3-L1 preadipocyte adipogenic differentiation at days 0, 2, 4, 6, and 8. Scale bar, 200 μm. **(B)** The *Lnc-FR332443* and *Runx1* mRNA levels during 3T3-L1 preadipocyte differentiation on different days. **(C)** Western blot analysis of Runx1 and adipogenic marker (anti-PPARγ and anti-C/EBPα) protein levels in 3T3-L1 preadipocytes differentiated on different days. **(D)** The quantification data of panel **(C)**. **(E)** The time course of *Lnc-FR332443* and *Runx1* mRNA expression during 3T3-L1 preadipocyte differentiation after infection with lentivirus-Lnc-FR332443. **(F)** Western blots of Runx1 protein expression in the 3T3-L1 preadipocytes differentiated on different days after lentivirus-Lnc-FR332443 infection. **(G)** The quantification data of panel **(F)**. All the data are shown as the mean ± SEM, NS denotes not significant, *N* = 3, *P < 0.05, **P < 0.01, ***P < 0.001, and ****P < 0.0001.

Subsequently, we designed *in vitro* experiments to confirm the role of *Lnc-FR332443* in adipogenic differentiation. We induced mouse 3T3-L1 preadipocytes to differentiate into mature adipocytes, which showed increased oil red O staining on different days (Figure 4A). During induction of adipogenesis, *Lnc-FR332443* was decreased gradually, followed by downregulated *Runx1* mRNA expression (Figure 4B). Furthermore, Runx1 protein expression was decreased, on the contrary, adipogenic marker gene expression of *PPAR*γ and *C/EBP*α were all upregulated as the induction time increased (Figures 4C,D). To further demonstrate the relationship between *Lnc-FR332443* and *Runx1* in adipocyte differentiation, we used an expression vector to overexpress *Lnc-FR332443* in 3T3-L1 preadipocytes. As expected, overexpression of *Lnc-FR332443* upregulated the *Runx1* mRNA (Figure 4E) and protein levels (Figures 4F,G). These data suggested that expression of *Lnc-FR332443* is positively associated with that of *Runx1* during adipogenesis, and overexpression of *lnc-FR332443* increased the *Runx1* expression. These findings indicated that *Lnc-FR332443* might target *Runx1* during adipogenic differentiation.

### *Lnc-FR332443* Inhibits 3T3-L1 Preadipocyte Differentiation

**FIGURE 5:**
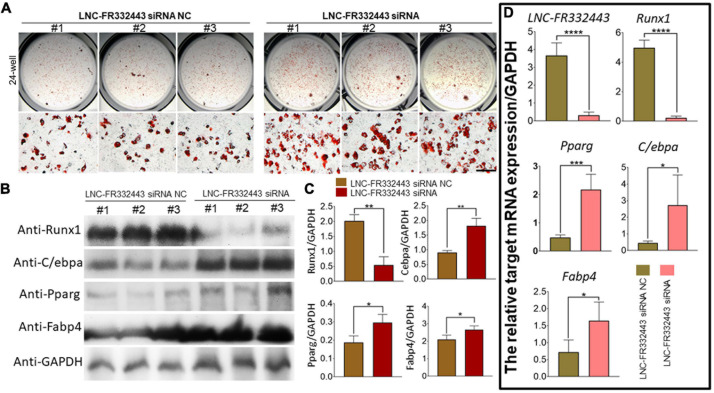
*Lnc-FR332443* inhibits 3T3-L1 preadipocyte differentiation *in vitro*. **(A)** Oil red O staining of 3T3-L1 preadipocytes transfected with *Lnc-FR332443* siRNA and its control at day 4. Scale bar, 200 μm. **(B)** The protein expression levels of Runx1 and the adipogenic markers PPARγ, C/EBPα and FABP4 in the 3T3-L1 cells transfected with *Lnc-FR332443* siRNA and its control. **(C)** Quantification data of panel **(B)**. **(D)** The mRNA expression levels of *Lnc-FR332443, Runx1, PPAR*γ, *C/EBP*α and *FABP4* in the 3T3-L1 cells transfected with *Lnc-FR332443* siRNA and its control. All data are presented as the mean ± SEM, NS denotes not significant, *N* = 4, *P < 0.05, **P < 0.01, ***P < 0.001, and ****P < 0.001.

To explore the role of *Lnc-FR332443* during adipogenesis *in vitro*, we first used an RNA interference technique to eliminate *Lnc-FR332443* expression during 3T3-L1 preadipocyte differentiation. Successful knockdown of *Lnc-FR332443* dramatically promoted the adipogenic differentiation of 3T3-L1 preadipocytes, characterized by increased oil red O staining (Figure 5A), and upregulated the expression of the adipogenic marker genes of *PPAR*γ, *C/EBP*α and *FABP4* at both protein level (Figures 5B,C) and mRNA level (Figure 5D). Surprisingly, *Lnc-FR332443* knockdown led to a decrease in the *Runx1* protein (Figures 5B,C) and mRNA levels (Figure 5D). Taken together, these data indicated that *Lnc-FR332443* plays a negative regulatory role in the process of adipogenic differentiation, which might be achieved by targeting *Runx1*.

### The Overexpression of *Runx1* Suppressesed 3T3-L1 Preadipocyte Differentiation

**FIGURE 6:**
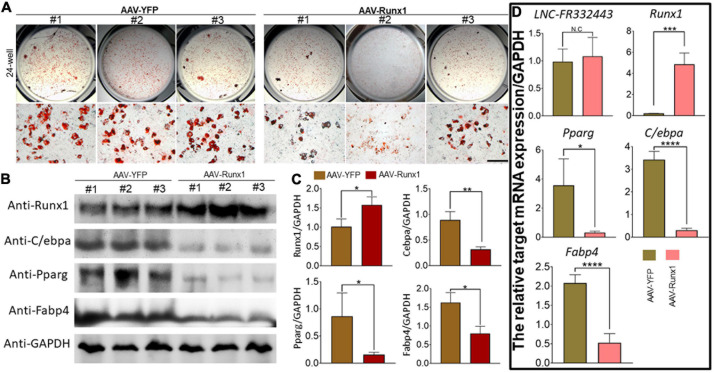
*Runx1* overexpression inhibits 3T3-L1 preadipocyte differentiation. **(A)** Oil red O staining of 3T3-L1 cells transfected with AAV-*Runx1* and its control at day 4. Scale bar, 200 μm. **(B)** The protein expression levels of Runx1 and the adipogenic markers PPARγ, C/EBPα and FABP4 in the 3T3-L1 cells transfected with AAV-*Runx1* and its control. **(C)** Quantification data of panel **(B)**. **(D)** The mRNA expression levels of *Lnc-FR332443, Runx1, PPAR*γ, *C/EBP*α and *FABP4* in the 3T3-L1 cells transfected with AAV-*Runx1* and its control. All data are presented as the mean ± SEM, *N* = 6, NS denotes not significant, *P < 0.05, **P < 0.01, ***P < 0.001, and ****P < 0.001.

To further explore the relationship between *Lnc-FR332443* and *Runx1*, we overexpressed *Runx1* in 3T3-L1 preadipocytes by adenovirus. After *Runx1* was overexpressed, the oil red O staining results showed that adipogenic differentiation was obviously inhibited in the AAV-*Runx1*-overexpressing groups compared to the control groups (Figure 6A). qRT-PCR and Western blotting were performed to evaluate the expression of *Runx1* in adipogenic differentiation. The overexpression of *Runx1* downregulated the protein and mRNA levels of the adipogenic marker genes *PPAR*γ, *C/EBP*α, and *FABP4* (Figures 6B,C), whereas *Lnc-FR332443* expression was not significantly changed (Figure 6D). These results suggested that the inhibitory effect of *Lnc-FR332443* on adipogenic differentiation is mediated by modulating the expression of *Runx1*. In contrast, the expression of *Lnc-FR332443* was not affected by *Runx1*.

### *Lnc-FR332443* Inhibited Adipogenesis by Downregulating the Expression of p38 and ERK1/2 and the Working Model of *Lnc-FR332443* in Regulating Adipogenesis

**FIGURE 7:**
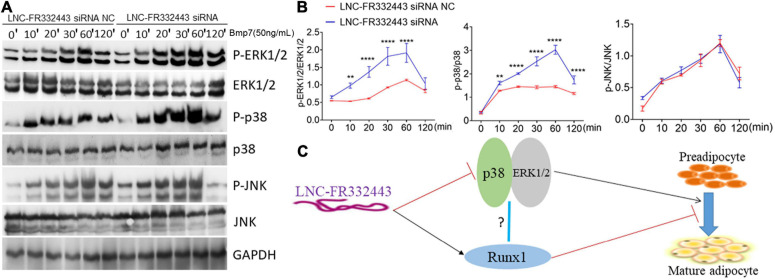
*Lnc-FR332443* inhibits 3T3-L1 cell differentiation by attenuating the phosphorylation of p38 and ERK1/2. **(A)** The phosphorylation levels of P-p38, P-ERK1/2 and P-JNK of the MAPK signaling pathway during 3T3-L1 preadipocyte differentiation with *Lnc-FR332443* knockdown. **(B)** Quantification data of panel **(A)**. **(C)** A working model of the role of *Lnc-FR332443* in the negative regulation of adipogenesis through *Runx1*, MAPK-p38 and MAPK-ERK1/2. All the data are presented as mean ± SEM, *N* = 3, **p < 0.01, ****p < 0.001.

Subsequently, we further explored the potential molecular mechanism underlying the function of *Lnc-FR332443* in regulating adipogenesis. Our previous RNA-seq analysis results showed that MAPK signaling pathway was involved in *Runx1* deficient osteoblasts within upregulated adipogenesis (Tang et al., 2021). Another research also showed that the MAPK signaling pathway involving p38 and ERK1/2 plays crucial positive roles in adipogenesis (Xu et al., 2014). Therefore, after the expression of *Lnc-FR332443* was knocked down with *Lnc-FR332443* siRNA in 3T3-L1 preadipocytes, the phosphorylated levels of p38 and ERK1/2 were significantly increased compared to those in the control preadipocytes, but the phosphorylated JNK expression had no significant differences (Figures 7A,B). This finding indicated that *Lnc-FR332443* inhibits adipogenesis by attenuating the expression of phosphorylation of p38 and ERK1/2 in the MAPK signaling pathway. Collectively, all these data in our research suggested a working model in which *Lnc-FR332443* inhibits adipogenesis by downregulating the expression of p38-MAPK and ERK1/2-MAPK signaling pathway as well as the expression of *Runx1* (Figure 7C).

## Discussion

Adipogenesis is a complicated process that involves many transcription factors and signaling pathways (Farmer, 2006). Recent transcriptomic analyses had revealed thousands of non-coding RNAs that could potentially regulate adipocyte development and differentiation at multiple levels (Engreitz et al., 2016; Ransohoff et al., 2018). Studies had shown that SRA in adipose tissue enhanced its transcriptional activity by binding to *PPAR*γ, thus promoting the differentiation of 3T3-L1 preadipocytes, and it could also regulate adipocyte differentiation through the MAPK and TNFα signaling pathways (Xu et al., 2010). In mature adipocytes, the DNA site of *Lnc-Leptin* could directly interact with *Lep* to maintain the expression of *Lep in vitro* and *in vivo*, thus promoting adipogenesis (Lo et al., 2018). In addition, a novel *adipoQ AS lncRNA* (the strands that were opposite to the previously annotated transcripts) that transfers from the nucleus to cytoplasm inhibits adipogenesis through the formation of an *AdipoQ AS lncRNA/AdipoQ* mRNA duplex to suppress the translation of *AdipoQ* mRNA (Cai et al., 2018). However, to date, the specific regulatory mechanism of lncRNAs in adipogenic differentiation was still unclear, and there were few lncRNAs that could be used as targets of anti-obesity drugs.

In this study, in order to identify lncRNAs/mRNAs selectively involved in adipocyte differentiation, we analyzed mWAT using microarray, and the data demonstrated that *Runx1*, which had been shown to negatively regulate adipogenesis in our previous research (Tang et al., 2021), was among the downregulated mRNAs in the F72h mice mWAT compared to the control mice. Further, we found *Lnc-FR332443*, which is the antisense LncRNA of *Runx1* through UCSC and Ensembl database search, was among the top 40 downregulated lncRNAs in the microarray analysis. Thus, we were the first group to identify *Lnc-FR332443* in response to external nutritional stimuli in mice stimulated by different nutritional conditions. We used adenovirus vectors to inject the adipose tissue in order to eliminate the expression of *Lnc-FR332443*, and then found the expression of *Lnc-FR332443* was dramatically decreased in mWAT, sWAT, and iBAT, but not in eWAT. In the following experiments, the weight of mWAT, sWAT, and iBAT were all higher in *Lnc-FR332443* injection group, but there was still no statistical weight difference in eWAT. The main cause was due to that the external *Lnc-FR332443* expression might activate the negative feedback mechanism in eWAT. In addition, a series of *in vitro* experiments were conducted to demonstrate that *Lnc-FR332443* could inhibit adipocyte differentiation. Surprisingly, the knockdown or overexpression of *Lnc-FR332443* in preadipocytes led to downregulated or upregulated *Runx1* expression, respectively. Taken together, these data suggested that *Lnc-FR332443* effectively inhibits fat formation by targeting *Runx1*.

Many studies had also focused on the function of *Runx1* in the developmental stage of skeleton (Yamashiro et al., 2004; Wang et al., 2005; Kimura et al., 2010), yet its role in adipocyte differentiation and fat formation and the mechanism underlying which factors regulate the preadipocyte to adipocyte lineage commitment remain unknown. Our previous research demonstrated that adipocyte differentiation was promoted when *Runx1* was deficient in chondrocytes and osteoblasts (Tang C.Y. et al., 2020; Tang et al., 2021). However, *Runx1* might be required to suppress adipocyte lineage commitment, which warrants further study to elucidate the underlying mechanisms. In this study, we found that *Runx1* was regulated by *Lnc-FR332443* in an epigenetic manner. By using both *in vitro* and *in vivo* studies with deletion of *Lnc-FR332443* expression in adipocyte tissue or cells, respectively, we found that the expression of *Runx1* was significantly downregulated with the knockdown of *Lnc-FR332443*. Likewise, the expression of *Runx1* was significantly upregulated by the overexpression of *Lnc-FR332443 in vitro*. Then, we overexpressed *Runx1* in 3T3-L1 preadipocytes to explore the upstream or downstream relationship between *Lnc-FR332443* and *Runx1*. As expected, the expression of *Lnc-FR332443* did not change significantly after *Runx1* was overexpressed. These data indicated that *Lnc-FR332443* positively regulates *Runx1* expression epigenetically during adipocyte differentiation. At present, there were many ways in which lncRNAs participate in the regulation of gene expression. For example, lncRNAs can recruit transcriptional regulators to adjacent target gene promoters, activate target genes by activating transcription factors, and enhance target gene transcription (Sun and Kraus, 2015). Second, lncRNAs can stabilize, promote or inhibit the translation of target mRNAs and even promote the decay of target mRNAs (Wei et al., 2016). In addition, lncRNAs can act as molecular baits to induce certain proteins, RNA or miRNA molecules to leave specific regions or act as “molecular sponges” to compete with miRNAs on mRNAs (Kameswaran and Kaestner, 2014). However, in this study, the in-depth molecular mechanism of how *Lnc-FR332443* regulates *Runx1* remains to be elucidated and may need to be proved in our further studies.

In addition, our results suggested that *Lnc-FR332443* represses adipocyte differentiation epigenetically by attenuating the phosphorylation of p38 and ERK1/2 in the MAPK signaling pathway. MAPK, the mitogen-activated protein kinases, is a group of evolutionarily conserved serine/threonine protein kinases that are activated by a series of extracellular signals and regulate many physiological activities, such as inflammation, apoptosis, carcinogenesis, tumor cell invasion, and metastasis (Chauhan et al., 1997; Han et al., 1997; Vo et al., 1998; Regalo et al., 2010). The MAPK signaling pathway is also involved in the functional regulation of adipose-derived stromal/stem cells (Imran et al., 2017; Wang et al., 2018; Fayyad et al., 2019). Recent studies had found that the long non-coding RNA *RUNX1-IT1* can inhibit hepatoma cell proliferation and promote hepatoma cell apoptosis by regulating the MAPK pathway (Yan et al., 2019). In our previous research, through RNA-seq analysis, we found that most of the MAPK signaling pathway genes expression were downregulated in *Runx1* deficient mesenchymal stem cells or mature osteoblasts in which the adipocyte commitment was increased (Tang et al., 2021), which showed that MAPK signaling pathway was closely associated with upregulated adipogenesis led by *Runx1* deficiency. However, it is unclear whether long non-coding RNAs can regulate adipogenesis by regulating the MAPK signaling pathway mediated by *Runx1*. In this study, we found that the expression of phosphorylated p38 and ERK1/2 at protein level increased significantly after *Lnc-FR332443* was eliminated, which meant p38 and ERK1/2, the important members of MAPK signaling pathway, can be the other potential targets of *Lnc-FR332443* besides for *Runx1*. However, whether p38 and ERK1/2 can be directly or indirectly regulated by *Lnc-FR332443* need to be more proved. On one hand that *Lnc-FR332443* is the antisense lncRNA of *Runx1*, but neither for p38 nor ERK1/2, on the other hand, in our previous research, it had been shown that the MAPK signaling pathway was involved in the upregulated adipogenesis mediated by *Runx1* deficiency (Tang et al., 2021). In addition, it had been found that *Runx1* could regulate the migration, invasion, and angiogenesis of human glioblastoma through the p38 MAPK pathway (Sangpairoj et al., 2017). The expression of *Runx* can antagonize the activation of p38 MAPK, a key mediator of ceramide-induced death (Kilbey et al., 2010). All of these previous research data demonstrated that *Runx1* might mediate the expression of p38 and ERK1/2 in adipocyte differentiation. Thus, *Lnc-FR332443* might also indirectly regulate p38 and ERK1/2 expression through *Runx1*, which still needs more experiments to be proved in our later studies.

In summary, our findings provided *in vivo* evidence that adipocyte-enriched lncRNAs *(Lnc-FR332443*) effectively suppress adipogenesis by targeting *Runx1* as well as p38-MAPK and ERK1/2-MAPK, which might provide a new therapeutic target for epigenetic drug intervention to combat the imminent epidemic of obesity and its related metabolic diseases.

## Data Availability Statement

The original contributions presented in the study are included in the article/Supplementary Material, further inquiries can be directed to the corresponding author/s.

## Ethics Statement

The animal study was reviewed and approved by the Ethics Committee of The Second Xiangya Hospital, Central South University.

## Author Contributions

H-DZ conceived and designed the study. FX, C-YT, H-NT, H-XW, NH, and LL performed experiments. FX, C-YT, H-NT, H-XW, NH, LL, and H-DZ analyzed data. FX and C-YT wrote the manuscript. All authors contributed to the article and approved the submitted version.

## Conflict of Interest

The authors declare that the research was conducted in the absence of any commercial or financial relationships that could be construed as a potential conflict of interest.

## Footnotes

1 www.geneontology.org

2 http://www.genome.jp/kegg/

3 http://genome.ucsc.edu

## Supplementary Material

Supplementary Figure 1 Verification of differentially expressed LncRNAs by qRT-PCR. Twelve differential expressed LncRNAs selected by co-expression and Venn analysis were further verified by qRT-PCR in control group, fasted for 72 h (72 h) group, and refeeding for 48 h (48 h) group. All data are expressed as the mean ± SEM, N.S denotes not significant, *N* = 6, *Indicates the difference between the control group and F72h. #Indicates the difference between F72h and R48h group **P* < 0.05, ***P* < 0.01, ****P* < 0.001, and *****P* < 0.0001. #*P* < 0.05, ##*P* < 0.01, ###*P* < 0.001, and ####*P* < 0.0001.

Supplementary Figure 2 The expression levels of *Lnc-FR265215* and *Lnc-FR067374* in various of tissues. **(A)** The expression level of *Lnc-FR265215* in various tissues of the mouse by qRT-PCR. **(B)** The expression level of *Lnc-FR067374* in various tissues of the mouse by qRT-PCR. All data are expressed as the mean ± SEM, *N* = 4.
