# Supplementary figures and images for: Long Non-coding RNA 332443 Inhibits Preadipocyte Differentiation by Targeting Runx1 and p38-MAPK and ERK1/2-MAPK Signaling Pathways
Source: Front Cell Dev Biol. 2021 Jun 8;9:663959. doi: 10.3389/fcell.2021.663959 (PMC8217766; doi:10.3389/fcell.2021.663959)

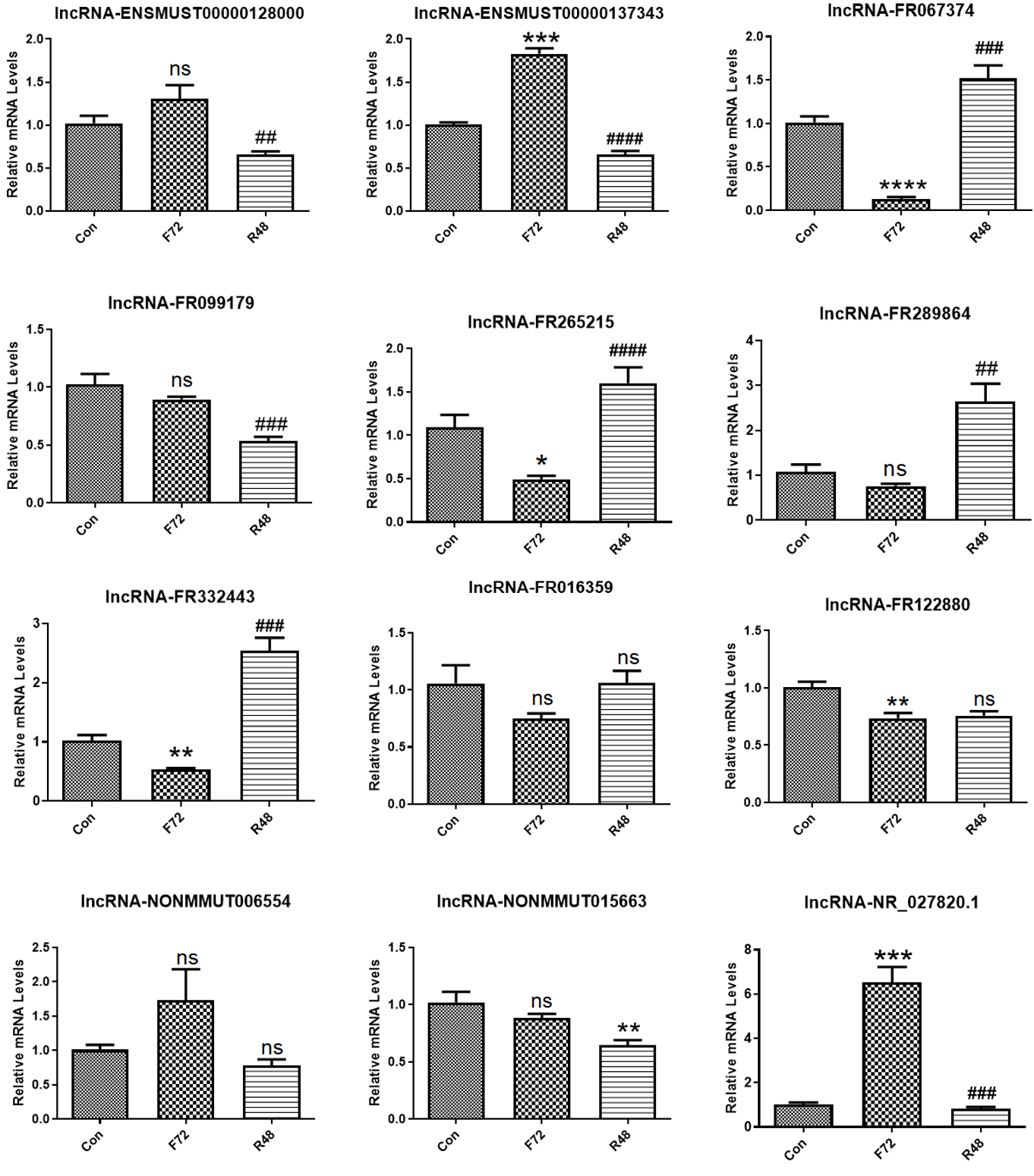

Supplement: Supplementary Figure 1 — Verification of differentially expressed LncRNAs by qRT-PCR. Twelve differential expressed LncRNAs selected by co-expression and Venn analysis were further verified by qRT-PCR in control group, fasted for 72 h (72 h) group, and refeeding for 48 h (48 h) group. All data are expressed as the mean ± SEM, N.S denotes not significant, N = 6, *Indicates the difference between the control group and F72h. #Indicates the difference between F72h and R48h group *P < 0.05, **P < 0.01, ***P < 0.001, and ****P < 0.0001. #P < 0.05, ##P < 0.01, ###P < 0.001, and ####P < 0.0001. [file Image_1.TIF]

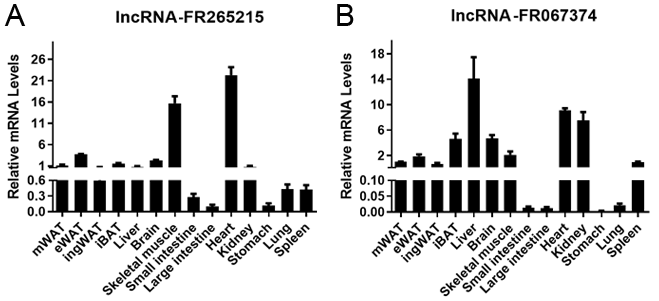

Supplement: Supplementary Figure 2 — The expression levels of Lnc-FR265215 and Lnc-FR067374 in various of tissues. (A) The expression level of Lnc-FR265215 in various tissues of the mouse by qRT-PCR. (B) The expression level of Lnc-FR067374 in various tissues of the mouse by qRT-PCR. All data are expressed as the mean ± SEM, N = 4. [file Image_2.TIF]
